# Prevalence of Fabry disease in male dialysis patients: Argentinean screening study

**DOI:** 10.1002/jmd2.12035

**Published:** 2019-05-02

**Authors:** Joaquín Frabasil, Consuelo Durand, Silvia Sokn, Daniela Gaggioli, Patricia Carozza, Ricardo Carabajal, Juan Politei, Andrea B. Schenone

**Affiliations:** ^1^ Laboratory of Neurochemistry “Dr. N. A. Chamoles” Buenos Aires Argentina; ^2^ Foundation for the Study of Neurometabolic Diseases (FESEN) Buenos Aires Argentina

**Keywords:** dialysis screening, dried blood spots, epidemiology, Fabry disease, prevalence

## Abstract

**Background:**

Fabry disease (FD) is an X‐linked lysosomal storage disorder caused by enzyme Alpha‐Galactosidase A (α‐Gal‐A) deficiency, due mutations in GLA gene. Progressive glycolipid accumulation leads to damage in kidney and other organs. The aim of this study was to estimate the prevalence of Fabry disease in Argentinean male patients undergoing dialysis.

**Methods:**

A prospective screening study was carried out measuring the α‐Gal‐A activity in dried blood spot (DBS) samples of male patients undergoing dialysis from Argentina. Those patients in which DBS α‐Gal‐A level was low (<4.0 μmol/hr/L), underwent GLA genetic testing for diagnosis confirmation.

**Results:**

Nine thousand six hundred and four dialysis male patients from 264 centers distributed over 20 of the 23 provinces of Argentina were investigated. Twenty‐four patients showed a decreased or absent α‐Gal‐A activity in DBS and although genetic analysis found a variant in the GLA gene in every one of these patients, we could confirm FD diagnosis in 22 cases.

**Conclusion:**

The prevalence rate of FD found in Argentinean male dialysis patients was 0.23%. Classic phenotype was observed in 73% of patients, whereas the remaining 27% presented as late‐onset variant. This was the largest study carried out in dialysis patients from a same country at a worldwide level and the first study performed in Argentina.

**SYNOPSIS**

The prevalence rate of FD found in Argentinean male dialysis patients was 0.23%.

## 1 INTRODUCTION

Fabry disease (FD, OMIM 301500) is a rare, progressive X‐linked lysosomal storage disease caused by mutations in the GLA gene encoding the acid hydrolase lysosomal enzyme Alpha‐Galactosidase A (α‐Gal‐A, EC 3.2.1.22),^1^, ^2^ which hydrolyses the terminal alpha‐galactosyl moieties from neutral sphingolipids.^3^ Deficiency or absence of enzymatic activity causes intracellular accumulation of globotriaosylceramide, galabiosylceramide, globotriaosylsphingosine (Lyso‐Gb3), and associated metabolites.^4^, ^5^

FD is characterized by a wide phenotypic spectrum, from mildly to severely affected male patients, and many manifestations are shared with other common diseases.^3^, ^6^ Typical severely affected male patients with the classic phenotype of the disease exhibit neuropathic pain in hands and feet, angiokeratomas, hypohidrosis, gastrointestinal dysfunction (abdominal cramps, diarrhea), and cornea verticillata as the initial signs and symptoms appearing in childhood or adolescence.^6^, ^7^ These are followed by progressive chronic kidney disease (CKD), rhythm and conductance disorders with progressive hypertrophic cardiomyopathy and stroke, complications occurring by the 3rd or 4th decade of life.^3^ Late‐onset variants present generally at an older age and show milder manifestations, in two distinct phenotypes: cardiac, that is typified by predominantly cardiac alterations; or renal, characterized by kidney damage principally.^3^, ^6^ Despite the X‐linked inheritance pattern, heterozygous females (HF) often show signs and symptoms of FD and may present phenotypes from asymptomatic to severe.^6^ This is thought to be related to a skewed X‐chromosomal inactivation.^8^

The true incidence of FD is difficult to establish and although it was estimated at 1 in ∼40 000‐60 000 males per year for the classic phenotype, more recent newborn screening programs found a frequency as high as 1 in 3100 live male births for the late‐onset variants.^8^ To date, more than 800 disease causing mutations have been reported.^9^

Regarding prevalence, more than 30 articles have been published worldwide on the screening of FD in patients receiving dialysis, suggesting that the prevalence may be higher in the dialysis population, where values of up to 1.2% have been reported.^9^, ^10^, ^11^, ^12^, ^13^, ^14^, ^15^, ^16^ However, none of these studies were performed in Argentina.

Since enzyme replacement therapy (ERT) became available, early detection of FD patients offers hope for altering the natural course of the disease.^8^ To this end, screening for FD in newborns and high‐risk patients (including those with chronic kidney disease or left ventricular hypertrophy, for example) has become a valuable approach for detecting FD patients.^8^, ^17^

The aim of this study was to estimate the prevalence of FD in Argentinean male patients undergoing dialysis. At the same time, we categorized patients in classic or late onset phenotype based on clinical parameters, residual α‐Gal‐A activity, genetic mutations and Lyso‐Gb3 plasma levels.

## 2 MATERIALS AND METHODS

### 2.1 Study design and subjects

We performed a prospective screening study from January 2012 to December 2016. Male patients undergoing dialysis from Argentina were included. Dried blood spot (DBS) samples were obtained prior to dialysis procedure in all cases. All patients that agreed to the screening were included, regardless of age and the clinical diagnosis of end stage renal disease (ESRD), except those previously diagnosed with FD. All patients provided signed written informed consent before inclusion, only 17 patients declined to consent for this study. The study was performed according to the principles of the Declaration of Helsinki and approved by the ethics committee of the Laboratory of Neurochemistry in Argentina. Women were not included in this study due to the high percentage of HF showing normal values of α‐Gal‐A activity in blood samples.^18^

The screening was carried out by a two‐step strategy. The first step was to measure the activity of α‐Gal‐A in DBS, using the enzyme Beta‐Galactosidase (β‐Gal) to evaluate the integrity of the sample. Those patients in which DBS α‐Gal‐A level was lower than reference range (4.0‐51.5 μmol/hr/L), and β‐Gal activity was between reference values (30.4‐128.4 μmol/hr/L), underwent GLA genetic testing for diagnosis confirmation. In some of these patients, but not all, we could measure plasma Lyso‐Gb3.

### 2.2 Measurement of α‐Gal‐A activity in DBS

The method applied is based on that published by Chamoles et al.^19^ Briefly, two 3.2 mm discs were obtained from every sample and placed individually in a well of a clear 96‐well microplate. A citrate‐phosphate pH 4.4 buffer, N‐acetyl‐D‐galactosamine, and 4‐methylumbelliferyl‐α‐D‐galactopyranoside (synthetic fluorogenic substrate) were added to each well. The mix was incubated for 20 hours at 37°C. After incubation, deproteinization with trichloroacetic acid and centrifugation were performed. Subsequently, supernatants were placed in a microplate for fluorometry containing a solution of ethylenediamine, and fluorescence reading was carried out using a microplate fluorometer (wavelength: excitation = 365 nm, emission = 450 nm).

### 2.3 Measurement of β‐Gal activity in DBS

Like α‐Gal‐A, the method applied is based on that published by Chamoles et al.^19^ Briefly, each 3.2 mm sampled disc was placed in a well of a clear 96‐well microplate. On each well a citrate‐phosphate pH 4.4 buffer, sodium chloride solution and 4‐methylumbelliferyl‐β‐D‐galactopyranoside (synthetic fluorogenic substrate) in water were added. The reaction was incubated for 3 hours at 37°C. Subsequently, deproteinization, centrifugation and fluorescence reading were performed, following the same method used for α‐Gal‐A activity measurement.

### 2.4 Measurement of Lyso‐Gb3 in plasma

Plasma Lyso‐Gb3 was measured in EDTA plasma samples. Briefly, Lyso‐Gb3 was extracted from plasma by solid phase (Waters Oasis McX). The separation and detection were done by ultra‐performance liquid chromatography system coupled with a tandem mass spectrometer (UPLC‐MS/MS) operated in MRM mode with a reverse phase column (Waters Corp. Milford, MA). Reference value for men was established as <1.0 nmol/L.

### 2.5 Bioinformatic analysis

Novel genetic variants were analyzed by bioinformatic predictors depending on mutation type. *In‐silico* tools used were: PolyPhen‐2 (http://genetics.bwh.harvard.edu/pph2) for missense mutations, PROVEAN (http://provean.jcvi.org/index.php) for missense and in‐frame deletions, and Human Splicing Finder (http://www.umd.be/HSF/) for intronic mutations. Mutation databases as The Human Gene Mutation Database (HGMD; http://www.hgmd.cf.ac.uk), Fabry-database.org (http://fabry-database.org/mutants/) and International Fabry Disease Genotype‐Phenotype Database (dbFGP; http://dbfgp.org/dbFgp/fabry) were consulted.

### 2.6 Genetic analysis

Genomic DNA was extracted from leukocytes using MasterPure DNA Purification Kit for Blood Version II (Epicentre, Wisconsin). Each exon and flanking intron sequence of the GLA gene was amplified by PCR using AmpliTaq gold 360 master mix (Applied Biosystems, Foster City, CA). PCR products were sequenced in both directions (forward and reverse) by the automated Sanger method (dideoxy terminators), using ABI Genetic sequencers (Applied Byosystems). Multiplex Ligation dependent Probe Amplification (MLPA, MRC Holland) was also performed in all cases.

### 2.7 Phenotype classification criteria

According to clinical manifestations, patients with confirmed FD were classified in classic form (Type 1) or late onset variant (Type 2). We considered affected males with classic form those patients who had childhood or adolescence onset of signs and symptoms of the disease, including angiokeratomas, acroparesthesias, hypohidrosis, cornea verticillata, gastrointestinal symptoms, cardiac and/or cerebrovascular manifestations, besides renal disease. On the other hand, were considered late onset patients those who only presented renal disease and lack of early manifestations of Type 1 phenotype.

### 2.8 Statistical analysis

Analysis was performed using STATA version 10.1 (Stata Corporation, TX). Baseline characteristics of the patients evaluated were reported as percentages for categorical data and mean with its SD for continuous data.

## 3 RESULTS

### 3.1 Screening for α‐Gal‐A deficiency

**Figure 1:**
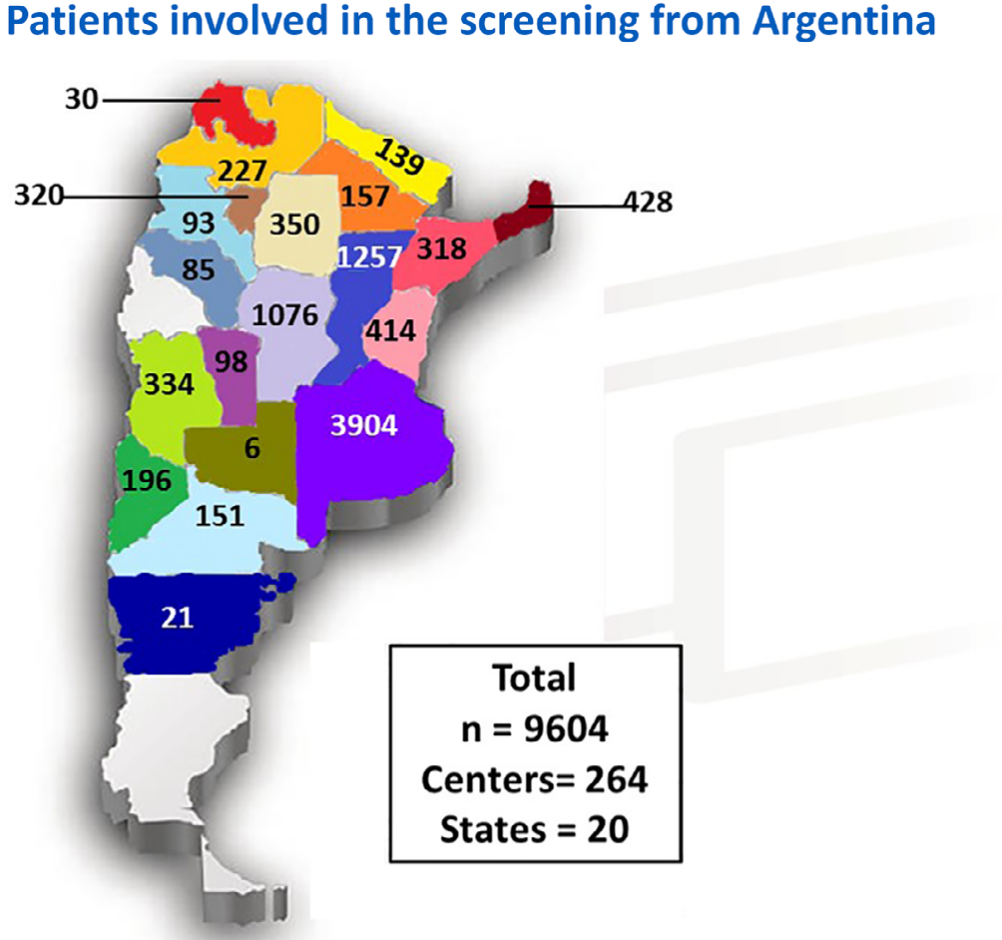
Distribution and number of dialysis patients in this screening study

The analysis of α‐Gal‐A activity in DBS was carried out in a total of 9604 dialysis male patients from 264 centers distributed over 20 of the 23 provinces of Argentina. Distribution across the country of this population is shown in Figure 1. The characteristics of analyzed population are summarized in Table 1. Mean age of the analyzed subjects was 33.7 years. In this first approach, 24 cases with decreased or absent α‐Gal‐A activity in DBS were found (Tables 1 and 2).

**Table 1:** Descriptions and summarized results of analyzed population

Population characteristics	
Patients analyzed (n)	9604
Mean age (y), SD	33.7 ± 29.8
Median age (y)	38
Range age (y)	18‐100
Patients with decreased α‐Gal A activity (n)	24
Confirmed FD patients (n)	22
FD patients α‐Gal A DBS activity (mean ± SD)	0.2 ± 0.17 μmol/hr/L
FD patients mean age (y), SD	44.0 ± 11.7
FD patients range age (y)	25‐67

Abbreviations: α‐Gal‐A, Alpha‐Galactosidase A; FD, Fabry disease; DBS, dried blood spots; m, months; SD, standard deviation; y, years.

**Table 2:** Characteristics and results of patients with decreased α‐Gal‐A activity

Patient ID	Age (y)	α‐Gal‐A activity in DBS (μmol/hr/L)	Gene analysis	Phenotype
1	25	0.1	p.C56S	Classic
2	38	0.2	p.L415P	Classic
3	41	0.0	c.640‐1G>C*	Classic
4	56	0.4	p.R363H	Late onset
5	33	0.1	p.L415P	Classic
6	54	0.2	p.L415P	Classic
7	32	0.2	p.G85D	Classic
8	46	0.2	c.886_887delAT*	Classic
9	43	0.2	p.L415P	Classic
10	43	0.0	Deletion exons 3‐4	Classic
11	40	0.1	Deletion exons 3‐4	Classic
12	54	0.0	c.1235_1236delCT (p.T412fs)	Classic
13	58	0.4	p.R363H	Late onset
14	59	0.3	p.R363H	Late onset
15	67	0.4	p.D109G	Late onset
16	41	0.4	p.W81X	Classic
17	50	0.4	p.P205S	Late onset
18	35	0.5	p.P409S	Classic
19	32	0.0	p.A156_A160del*	Classic
20	38	0.1	RNA analysis pending^a^	Classic
21	26	0.0	c.902_905insTGTC*	Classic
22	63	0.5	p.D109G	Late onset
23	42	0.8	p.D55G^b^, *	Non confirmed FD
24	19	1.8	p.G80D^c^	Non confirmed FD

α‐Gal‐A, Alpha‐Galactosidase A; DBS, dried blood spots; FD, Fabry disease; y, years.

α‐Gal‐A Activity in DBS reference value: ≥4.0 μmol/hr/L.

^*^Novel mutation.

a Plasma Lyso‐Gb3 (nmol/L): 23.6 (reference value <1.0).

b Plasma Lyso‐Gb3 (nmol/L): not detectable (reference value <1.0).

c Plasma Lyso‐Gb3 (nmol/L): 0.4 (reference value <1.0).

### 3.2 Genetic analysis and diagnosis confirmation

Genetic analysis was performed in all the 24 patients with decreased or absent α‐Gal‐A activity, finding a genetic variant in the GLA gene in all of them (Table 2). Sixteen different mutations were found, being five of them novel variants. This data, added to the clinical history, confirmed the diagnosis of FD in 22 of 24 patients. In one of the patients (ID 20, Table 2) gene sequencing (Sanger method and next generation sequencing) and MLPA did not reveal any genetic variant, but suggested us a possible mutation in intron 6 that could affect splicing, so we are currently studying these cases at RNA level (sequencing) to find the genetic cause of deficient α‐Gal‐A. However, clinical history, elevated plasma Lyso‐GB3 levels and relatives diagnosed with renal biopsies compatibles with FD, allowed us to confirm diagnosis of FD in this patient. In consequence, the prevalence rate of FD found in Argentinean dialysis patients was 0.23%.

We could not confirm FD in two patients with low α‐Gal‐A activity (patients ID 23 and 24, Table 2). Although a novel variant was found in one of these patients (ID 23), and a variant of unknown significance in the other (ID 24), the levels of plasma Lyso‐Gb3 were normal, and clinical history and detailed examination of these patients were not conclusive. Therefore, we considered them as likely benign variants causing pseudodeficient activity of α‐Gal‐A in DBS.

### 3.3 Fabry disease phenotypes

Sixteen of the 22 FD patients had the classic phenotype, and six patients with late‐onset variant were detected (Table 3). There was a significant difference between these groups in age at diagnosis (classic FD: mean age ± SD = 38.8 ± 8.4 years; late onset patients: mean age ± SD = 58.8 ± 5.8 years, *P* < .001) and in age at dialysis onset (classic FD: mean age ± SD = 37.4 ± 7 years); late onset patients: mean age ± SD = 56.5 ± 4.9 years, *P* < .001). We also found a significant difference in mean α‐Gal‐A activity between these two groups (0.14 ± 0.15 μmol/hr/L in classic FD vs 0.40 ± 0.06 μmol/hr/L in late onset variant, *P* < .001), but some overlap in a few cases was observed.

**Table 3:** Characteristics found in FD patients by phenotype

	Phenotype	
	Classic (Type I)	Late Onset (Type II)
n (%)	16 (73%)	6 (27%)
Age in years		
Mean ± SD	38.8 ± 8.4	58.8 ± 5.8
Range	25‐54	50‐63
α‐Gal‐A activity DBS (μmol/hr/L)		
Mean ± SD	0.14 ± 0.15	0.40 ± 0.06
Range	0.0‐0.5	0.3‐0.5
Mutation type		
Missense (% patients)	4 (43.8%)	3 (100%)
Nonsense (% patients)	1 (6.3%)	–
Deletion (% patients)	4 (31.3%)	–
Insertion (% patients)	1 (6.3%)	–
Consensus splice site (% patients)	1 (6.3%)	–
RNA analysis pending	1 (6.3%)	–

Abbreviations: α‐Gal‐A, Alpha‐Galactosidase A; DBS, dried blood spots; SD, standard deviation.

### 3.4 Analysis of novel variants by bioinformatic tools

Three of the five novel variants found, were able to be analyzed by bioinformatic predictor tools (results are shown in Table 4). The two remaining novel variants, c.886_887delAT and c.902_905insTGTC, were frameshift type mutations and can be considered as pathogenic variants, following the guidelines for the interpretation of sequence variants of the American College of Medical Genetics (ACMG).^26^ Additionally, the splice site variant c.640‐1G>C can be interpreted as pathogenic, according to the same guidelines.

**Table 4:** Analysis of novel variants by *in‐silico* predictive tools

Mutation	cDNA	PolyPhen‐2 Prediction (score)	PROVEAN prediction (score)	Human Splicing Finder 3.0 interpretation
p.D55G	c.164A>G	Benign (0.435)	Deleterious (−5.382)	–
p.A156_A160del	c.467_481del15	–	Deleterious (−32.948)	–
N/A	c.640‐1G>C	–	–	Alteration of the wild type acceptor site, most probably affecting splicing

## 4 DISCUSSION

During 5 years of study, 9604 patients undergoing dialysis in 264 centers of 20 provinces from Argentina were enrolled. As a result, 22 patients with FD were identified. Therefore, the prevalence of Fabry disease in Argentinian male dialysis patients was 0.23%. This is the largest study carried out in dialysis patients related to the number of samples analyzed and the number of diagnoses performed.

Previously reported studies showed a prevalence of FD in dialysis patients that ranged from 0 to 1.17% in European, Asian and North American populations^14^, ^15^, ^21^; however, few studies exist in Latin America.^14^, ^18^ Most of the publications did not describe the phenotype features, genetic analysis and/or plasma Lyso‐Gb3 levels, making a possible overestimation of FD diagnoses.^20^

Recently, a Japan FD screening study (J‐FAST) dealt with 8547 patients on dialysis.^15^ At the tertiary examination only 2 out of 8547 patients were found to be positive for FD; therefore, the prevalence of FD reported was 0.02%, leading the authors to the conclusion that FD could not be ruled out as the clinical diagnosis of ESRD. In another representative study performed in Italy, Spada et al. screened a total of 6378 male dialysis patients being the prevalence of FD 0.25%.^22^

Regarding information from Latin America, in Brazil Porsch et al. estimate the prevalence of FD among ESRD males undergoing hemodialysis in Rio Grande do Sul, the southernmost state of Brazil.^14^ In a total of 548 male patients evaluated, two cases of FD were found, giving a prevalence of 0.36%.^14^ In another study carried out in Brazil by Silva et al, three cases of FD were found from a total of 2583 male screened patients, reporting a prevalence of 0.12% in Bahia State.^20^

Prevalence found in our study is in line with that reported by Spada et al and falls between those observed in Brazil and other Latin American countries, including Peru (0.3%) and Colombia (0.4%).^14^, ^20^ These comparable findings could be partly attributed to the ethnic profile of these countries, considering that the southern Brazilian population is predominately shaped by European descent, as is the case of Argentina.^14^, ^20^

Fabry phenotype can be defined based in age of onset of symptoms, clinical manifestations, residual enzyme activity, plasma Lyso‐Gb3 level and distribution of substrate deposition in different tissues.^16^ Males with classic phenotype generally experience the onset of symptoms at an earlier age than females with classic FD (median age: 6 years in males vs 9 years in females).^8^, ^16^ The most frequent symptoms reported in children with classic phenotype were neuropathic pain, followed by abdominal pain, diarrhea and angiokeratomas.^23^ In contrast, late onset variants, develop symptoms during the fourth or fifth decade of life without the presence of pain, diarrhea or skin manifestations.^13^ Residual enzyme activity level correlates with the phenotype where classic variant shows absence or severe deficiency (<1% of normal range) and late onset keeps between 3 and 30% of normal values.^13^

Currently, plasma Lyso‐Gb3 might be the most useful biomarker for FD.^21^ Males with classic or late onset phenotype show high levels of plasma Lyso‐Gb3, even when higher levels are reported in classic variant, all male patients present levels above the normal range.^16^ On the other hand, although females with classic phenotype always present plasma Lyso‐Gb3 levels above the normal range (though not so high as in males), normal levels in females with late onset phenotype has been reported, being the only limitation for this biomarker.^16^

Concerning phenotype, we found that 73% of the patients presented with classic FD, whereas the remaining 27% presented with late‐onset phenotype. A significant statistical difference related to the age at diagnosis, dialysis onset and residual enzymatic activity between these two phenotypes, were found in our population.

Recently, Doheny et al reanalyzed the prevalence of FD reported in screening studies performed in different high‐risk populations groups (dialysis, renal transplant, cardiac and stroke patients).^24^ They found that removing the benign and likely benign variants from each of the 63 screening studies that reported *GLA* mutations, previously published estimated prevalences were decreased in all the groups.^24^ Regarding dialysis patients, they found that among the 23 954 screened patients, 50.5% of the diagnoses performed in those studies had benign variants. When they corrected the available data by removing these nondisease‐causing variants, the prevalence of FD in male hemodialysis patients was reduced to 0.21%, of which 66% of the total cases were classic forms and 34% late onset forms. These results are very similar to those found in our study.

Regarding the novel variants found in our study, mutation c.640‐1G>C maps in the splicing acceptor site of intron 4 and the bioinformatic tool Human Splicing Finder predicted that it probably affects splicing due to the breakdown of this consensus sequence. Databases revealed that there is a variant previously reported as pathogenic in this position, c.640‐1G>A.^25^ Furthermore, this mutation can be interpreted as deleterious according to the ACMG variant interpretation guidelines.^26^ Variant p.A156_A160del is caused by an in‐frame deletion of 15 nucleotides (c.467_481del15) with the concomitant loss of 5 amino acids (A156, Q157, T158, F159 and A160). When this variant was analyzed with PROVEAN tool, the prediction made was deleterious. Mutation c.886_887delAT produces a truncated protein due to the loss of two nucleotides and the frameshift occasioned at exon 6. In the same exon, the insertion of four nucleotides in the variant c.902_905insTGTC produces a similar result, a truncated protein.

About plasma Lyso‐GB3, we have data from two of the patients with novel mutations (ID 19 and 21), but although both have elevated levels of this biomarker, samples were unfortunately collected after the beginning of treatment. On the other hand, from the two remaining patients with novel variants and confirmed FD (ID 3 and 8), we have plasma Lyso‐GB3 levels from some FD relatives of each of them, and levels were elevated in all cases.

As previously mentioned, in two cases we could not confirm diagnosis of FD. In the first case, we considered novel variant p.D55G as a likely benign mutation. Clinical and biochemical findings obtained here (plasma Lyso‐Gb3 was between reference value) do not allow us to classify this variant as pathogenic. Regarding bioinformatic tools opposite results were observed. Polyphen‐2 predicts it as a benign variant, whereas PROVEAN predicts it as a deleterious variant (Table 4), but this situation can be explained considering that predictors have an average accuracy around 80%.^27^ There is no other disease‐causing mutation reported in this residue in any of the databases. In the second case, variant p.G80D was reported in only one publication^11^ where they found three hemizygous individuals with normal to slightly elevated plasma Lyso‐Gb3 values. This variant is reported in HGMD and dbFGP database where classification is already unclear since it is considered as a likely late‐onset or benign variant. Clinical and biochemical findings in our case cannot confirm FD.

A possible limitation of our study is the noninclusion of all dialysis centers in the country. However, many patients were included, covering more than 50% of male dialysis patients in the country and therefore limiting recruitment bias (total number of patients in dialysis in Argentina during period of this study: 28960, where 62% were men, data from the Confederation of Dialysis Associations of the Argentine Republic). Another limitation regarding new genetic variants found of confirmed FD patients is the fact that it was not possible for us to measure plasma Lyso‐Gb3 or perform renal biopsies for these patients. Despite novel variants from confirmed FD patients interpreted as pathogenic following ACMG guidelines,^26^ it would be important to have full characterization by functional enzyme studies and biomarker testing in such cases.

## 5 CONCLUSIONS

We estimate a prevalence of 0.23% of FD in male patients undergoing dialysis in Argentina. We found that 73% of patients diagnosed had the classic phenotype, whereas the remaining 27% presented with late‐onset variant. This is the largest study carried out in dialysis patients from a same country and the first study performed in Argentina, contributing to fill the epidemiological gap of the disease in the country as well as in the South American region. Results reported here clearly show the value of carrying out screening studies of FD in dialysis patients and, at the same time, the need of a careful phenotype classification and interpretation of genetic analysis, for ruling out the presence of benign variants.

## CONFLICT OF INTEREST

Joaquin Frabasil declares received honoraria for lectures on Fabry disease from Sanofi Genzyme.

Consuelo Durand declares no conflict of interest.

Silvia Sokn declares no conflict of interest.

Daniela Gaggioli declares no conflict of interest.

Patricia Carozza declares no conflict of interest.

Ricardo Carabajal declares no conflict of interest.

Juan Politei declares received honoraria for lectures on Fabry disease from Sanofi Genzyme, Shire HGT, Amicus, Actelion, and Protalix.

Andrea Schenone declares received honoraria for lectures on Fabry disease from Sanofi Genzyme.

## AUTHOR CONTRIBUTIONS

J.F. and A.S. contributes with study design, sample acquisition, data analysis, and manuscript development.

C.D., S.S., D.G., P.C., R.C., and J.P. contributes with sample acquisition and manuscript development.
